# Exposure to hot and cold environmental conditions does not affect the decision making ability of soccer referees following an intermittent sprint protocol

**DOI:** 10.3389/fphys.2014.00185

**Published:** 2014-05-20

**Authors:** Lee Taylor, Natalie Fitch, Paul Castle, Samuel Watkins, Jeffrey Aldous, Nicholas Sculthorpe, Adrian Midgely, John Brewer, Alexis Mauger

**Affiliations:** ^1^Sport Science and Physical Activity, Sport and Exercise Science Laboratory, University of BedfordshireBedford, UK; ^2^School of Science, University of West ScotlandHamilton, Scotland; ^3^Department of Sport and Physical Activity, Edge Hill UniversityOrmskirk, UK; ^4^School of Sport and Exercise Sciences, University of KentChatham Maritime, UK

**Keywords:** soccer referee, thermoregulation, cognition, hot, cold

## Abstract

Soccer referees enforce the laws of the game and the decisions they make can directly affect match results. Fixtures within European competitions take place in climatic conditions that are often challenging (e.g., Moscow ~ −5°C, Madrid ~30°C). Effects of these temperatures on player performance are well-documented; however, little is known how this environmental stress may impair cognitive performance of soccer referees and if so, whether exercise exasperates this. The present study aims to investigate the effect of cold [COLD; −5°C, 40% relative humidity (RH)], hot (HOT; 30°C, 40% RH) and temperate (CONT; 18°C, 40% RH) conditions on decision making during soccer specific exercise. On separate occasions within each condition, 13 physically active males; either semi-professional referees or semi-professional soccer players completed three 90 min intermittent treadmill protocols that simulated match play, interspersed with 4 computer delivered cognitive tests to measure vigilance and dual task capacity. Core and skin temperature, heart rate, rating of perceived exertion (RPE) and thermal sensation (TS) were recorded throughout the protocol. There was no significant difference between conditions for decision making in either the dual task (interaction effects: FALSE *p* = 0.46; MISSED *p* = 0.72; TRACKING *p* = 0.22) or vigilance assessments (interaction effects: FALSE *p* = 0.31; HIT *p* = 0.15; MISSED *p* = 0.17) despite significant differences in measured physiological variables (skin temperature: HOT vs. CONT 95% *CI* = 2.6 to 3.9, *p* < 0.001; HOT vs. COLD 95% *CI* = 6.6 to 9.0, *p* < 0.001; CONT vs. COLD 95% *CI* = 3.4 to 5.7, *p* < 0.01). It is hypothesized that the lack of difference observed in decision making ability between conditions was due to the exercise protocol used, as it may not have elicited an appropriate and valid soccer specific internal load to alter cognitive functioning.

## Introduction

Soccer is considered the most popular sport in the world (Reilly, 1997), with elite competitive matches requiring an officiating team [referee, assistants × 2 and within the Union of European Football Associations (UEFA) region 2 × goal line officials] to apply the laws of the game (Castagna et al., 2007). Football is characterized by variable intermittent activity and recovery, inherently displaying more complex physiological requirements than sports with continuous exercise (Drust et al., 2000). The physiological demands of refereeing are similar to that of a midfield soccer player, with total distance covered and high speed running consistent between player and referee (Weston et al., 2011).

Increased physical fatigue in a referee can elicit an inability to maintain proximity to key incidents within game play (varying rule infringements), with increases in the infringement/distance nexus, known to impair decision making ability (Catterall et al., 1993). Therefore, repeated sprint ability is of high importance for referees to maintain proximity to such incidents (Galanti et al., 2008). Compounding issues regarding proximity, high intensity exercise *per se* is known to have negative effects on cognition (McMorris and Graydon, 1997), with such cognitive decrements associated with altered arousal and narrowed attention (Brisswalter et al., 2002). Thus, the interplay between the physical demands of refereeing and decision making is important, yet, requires further elucidation.

The UEFA Champions League and The UEFA Europa League, played principally in Europe between September and May, ensures referees often perform their roles within varying challenging environmental conditions [Madrid (~30°C) and Moscow (~ –5°C)] within the UEFA region and season. Indeed, intermittent sprint exercise in hot conditions (exercise-heat stress) is known to increase thermoregulatory and physiological strain/load (Duffield et al., 2009), reducing physiological performance (i.e., distance covered), and likely further disturbs arousal levels (Bandelow et al., 2010), when compared to similar exercise in temperate conditions. Conversely, cold temperatures can cause severe discomfort and lead to a drop in body temperature (Parsons, 2003), which, may also effect cognitive performance due to distraction (Mäkinen et al., 2006). However, metabolic heat production during soccer specific exercise may help overcome the distraction and severe discomfort associated with cold temperatures (Brotherhood, 2008), although, whether this ameliorates the well-reported negative effects on cognitive function (Mäkinen et al., 2006; Racinais et al., 2008) has yet to be confirmed. Despite evidence that both heat (Racinais et al., 2008; Simmons et al., 2008) and cold stress (Mäkinen et al., 2006) detrimentally effect cognitive capacity and performance, this has not been studied in soccer referees, who are constantly required to make correct decisions on infringements and are regularly exposed to environmental extremes within the UEFA region and season, whilst, performing soccer specific exercise. Referee decisions can often affect the outcome of a match and thus it is important to understand the interaction between the ambient environment and the ability of soccer referees to make correct decisions.

Due to inherent difficulties, and likely confounding nature in the inability to standardize the external environment (both weather and games factors players, tactics, results, etc.) and physiological demands within an actual match the tripartite research questions of interest here (decision making, physiological demands/capacity and environmental fluctuations) would be best explored by a treadmill based, environmental chamber delivered protocol. The seminal work of Drust et al. (2000) is currently the most used protocol and will be utilized within the present study.

The aims of the present study were to investigate the effect of hot and cold conditions, typical of the UEFA region and season, on decision making and physiological responses of soccer referees during a treadmill based intermittent sprint protocol (Drust et al., 2000). It was hypothesized that hot temperatures would increase the fatigue that is associated with the exercise protocol and thus cause a detrimental effect on decision making. Cold conditions would have no significant effect on core temperature, but skin temperature (*T*_sk_) would significantly decrease, resulting in a negligible effect on decision making ability.

## Methods

### Subjects and general experimental controls

Thirteen physically active males; either semi-professional referees (*n* = 7) or semi-professional soccer players (*n* = 6) (mean ± *SD*: age = 20.3 ± 2.2 years; height = 175 ± 7.4 cm; body mass = 70.8 ± 4 kg) volunteered for this study. Sample size of 12 was calculated using computer software (G^*^Power 3), and was deemed sufficient to observe significant differences in the dependant variable rectal temperature (*T*_re_) based on the previous findings (Drust et al., 2000). All participants were fully informed of the risks associated with this study before they gave full written consent to take part in testing. The procedures were approved by the University of Bedfordshire Ethics Committee. All participants abstained from alcohol, cigarettes, caffeine and strenuous exercise at least 48 h prior to testing and maintained their normal diet prior to and during the testing sessions (in line with; Taylor et al., 2012). Additionally, all participants refrained from supplementation of ergogenic aids throughout the study and abstained from exposure to extreme hot or cold conditions 7 d prior to testing (in line with; Taylor et al., 2012). Adherence was assessed by questionnaire, with no violations seen for these control parameters.

A urine refractometer (Alago Vitech Sicentific, Pocket PAL-OSMO, West Sussex, UK) was used to measure the hydration levels of the participants on arrival of each visit. Participants were asked to consume ~5–7 ml^·^kg^−1^ of water 2–3 h before each experimental visit, in line with previous recommendations (Sawka et al., 2007). A participant was deemed to be euhydrated if urine osmolarity was < 600 mOsm·Kg^−1^ H_2_O as previously used (Hillman et al., 2011), this control was not violated for any experimental procedure or exercise bout.

### Procedures

Participants reported to the laboratory on five occasions. The first two were to collect anthropometric data and for familiarization to the intermittent sprint exercise protocol (Drust et al., 2000) and cognitive tests (Hope et al., 1998). Visit 1 consisted of familiarization to the cognitive software, where as visit 2 included familiarization to both the cognitive software and the intermittent sprint protocol. For the remaining three visits, which were each separated by 7 d, participants completed the protocol in a randomized order inside an environmental chamber in either a cold condition [COLD; −5°C; 50% relative humidity (RH)], temperate control condition (CONT; 18°C; 50% RH) or hot condition (HOT; 30°C; 50% RH).

### Visit 1

#### Anthropometric data and familiarization

Upon arrival to the laboratory, air displacement plethysmography (BodPod, 2000A, Cranlea, Birmingham, UK) was used to assess body composition in accordance with the manufacturers' guidelines.

***Cognitive tests.*** Participants sat in the environmental chamber at a temperature of 18°C and 50% RH and performed the vigilance (VIG) and dual task (DT) cognitive tests, using the Psyche software package (Hope et al., 1998). To ensure all learning effects were removed, participants were familiarized to both tests on two occasions (Hope et al., 1998). The VIG test measured vigilance performance which consisted of 3 digit numbers that flashed on a screen 100 times per min. Participants were required to press the space bar on a keyboard when they identified that the 3 digit number was duplicated (Hope et al., 1998). FALSE, HIT, and MISSED scores were recorded (see Table 1). The DT test measures tracking and visual reaction time. To measure tracking, participants were required to control the screen cursor which must follow a moving blue circle on the computer screen. At random intervals, a small icon appeared on the screen and the participant had to press the spacebar to acknowledge the icon (Hope et al., 1998). FALSE, MISSED, and TRACKING scores were recorded (see Table 1). Both tests lasted for 3 min.

**Table 1:** **Ecological examples of cognitive tests used**.

**Output**	**Vigilance**		**Dual task**	
	**Definition**	**Ecological example**	**Definition**	**Ecological example**
FALSE	Recorded when a numerical duplication is incorrectly acknowledged.	A referee awards a free kick to the attacking team when the attacking player has dived.	When the participant incorrectly indicates the icon is present when it is not.	Referee incorrectly indentifies an off the ball incident (in their field of vision), whilst concentrating on the ball.
MISSED	Recorded when a numerical duplication is missed.	A referee fails to spot an infringement e.g., a player is fouled but no free kick awarded.	When the participant fails to indicate the presence of the icon.	Referee fails to spot an off the ball incident which has taken place close to the ball (in their field of vision).
HIT	Recorded when a numerical duplication is correctly identified.	When a referee correctly indentifies an infringement and a free kick is awarded.	N/A	N/A
TRACKING	N/A	N/A	The participant's ability to track a moving target around the screen with the mouse cursor.	Referees ability to track the ball e.g., a corner is taken and the ball enters the penalty area and has its trajectory diverted quickly.

***Exercise protocol.*** A warm-up, intermittent sprint protocol and cool down were all conducted on a motorized-treadmill (Woodway, PPS55 Med-I, Cranlea, Birmingham, UK) as a familiarization to the exercise protocol. Participants completed a 5 min warm up which consisted of jogging at a speed of 10 km.h^−1^ at 1% gradient. They then completed 45 min of the intermittent exercise protocol (Drust et al., 2000) to experience the changes of intensities and general requirements of successful protocol completion. The full 90 min protocol (Drust et al., 2000) was not used for familiarization due to both halves using the same movement patterns. Core temperature (rectal temperature) and Tsk were recorded during familiarization in order for participants to experience the full data collection process. This was completed in temperate conditions (18°C, 40% RH).

#### The intermittent sprint exercise protocol

**Figure 1:**
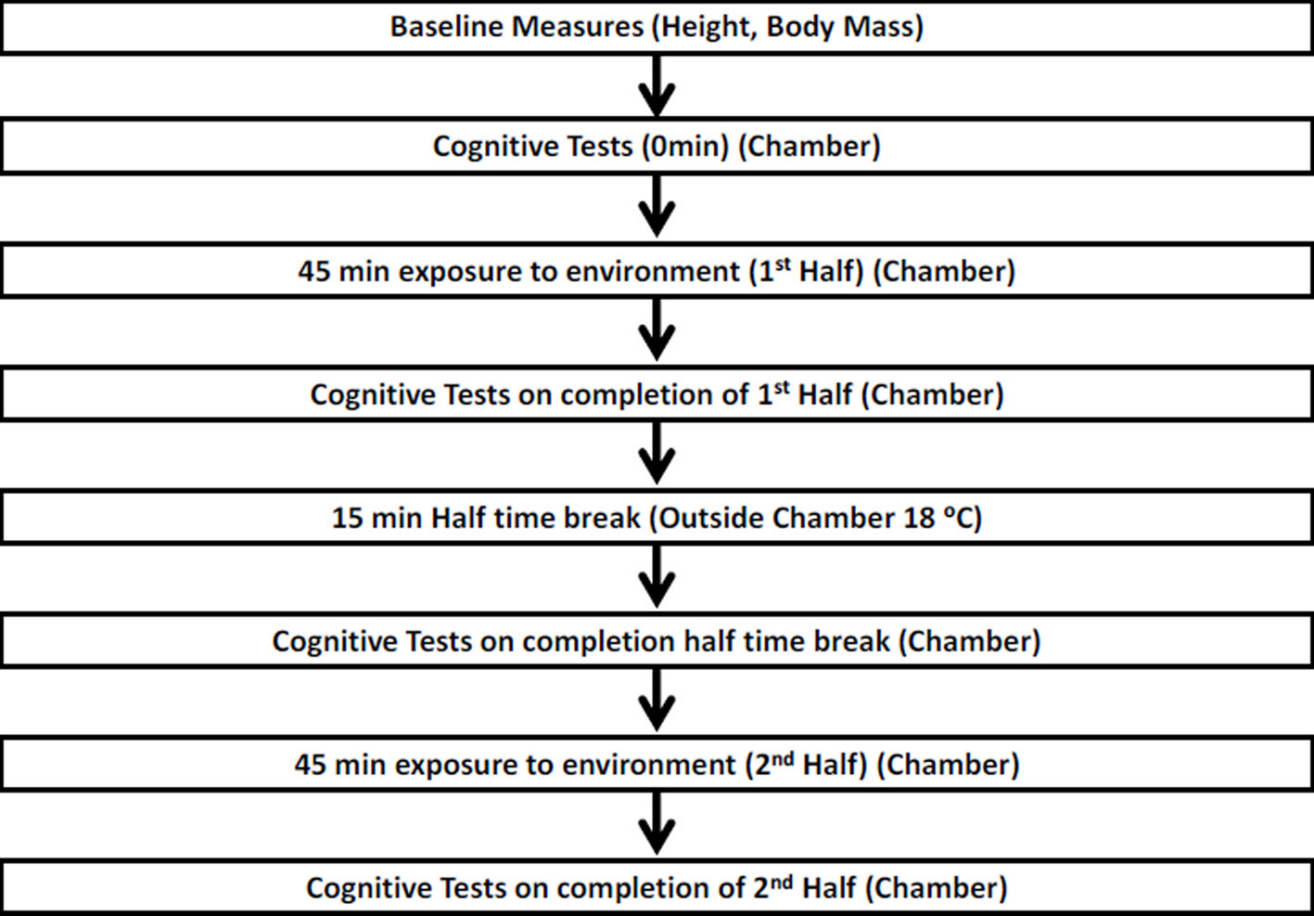
**Schematic of the 90 min experimental procedure which was carried out in all 3 conditions**.

A 105 min protocol including 90 min of exercise (composed of 2 × 45 min halves of intermittent exercise, interspersed with a 15 min rest period) was utilized (Drust et al., 2000), which, replicated the movement patterns and thus physiological loads experienced by referees during a football match (Figure 1). As professional referee movement patterns are similar to those of a soccer midfielder (Weston et al., 2011) the use of a protocol designed to mimic the movement patterns of an outfield player is justified. A timeline of the protocol is shown in Figure 1.

The 90 min of exercise consisted of 5 movements that are observed during a match. These include standing, walking, jogging, running and sprinting. The duration and speeds are shown in Table 2.

**Table 2:** **Speed and durations of the 5 different movements during the 90 min intermittent sprint exercise protocol**.

**Movement**	**Speed (km.h^**−1**^)**	**Duration (s)**	**Movements in half**
Stand	0	60	7
Walk	6	120	7
Jog	12	57	19
Run	15	33	8
Sprint	20	6	6

The varying speeds were randomly assigned to different time points of the 90 min protocol i.e., varying speeds were randomly assigned throughout the master protocol which was used for all subjects in all conditions.

### Experimental design

Participants were asked to wear t-shirt and shorts for testing conditions, in line with referee's attire during a match.

Once hydration status had been assessed, a heart rate (HR) monitor (Polar, FS1, Cranlea, Birmingham, UK) was attached to the chest. A rectal thermistor (Henleys, 400H and 4491H, Henleys, Herts, UK) inserted 10 cm past the anal sphincter was used to measure *T*_re_, with *T*_re_ recorded via a temperature monitor (Libra Medical, ET402, Cranlea, Birmingham, UK). *T*_sk_ was assessed using skin thermistors (Grant, EUS-U-VS5-0, Wessex Power, Dorset, UK) which were placed on the pectoral, tricep, gastrocnemius and vastus lateralis of the right side of the body (Ramanathan, 1964) using general medical tape. Once all thermistors were attached, they were connected to a data logger to record *T*_sk_ (Squirrel 451, Grant instruments, Wessex Power UK). Mean *T*_sk_ was calculated using the following equation (Ramanathan, 1964):

$$\begin{array}{l} \text{Mean Skin Temperature }(T_{\text{sk}}):0.3T_{\text{chest}}+0.3T_{\text{arm}}\\ \;+0.2T_{\text{thigh}}+0.2T_{\text{calf}} \end{array}$$

Once all baseline measurements were recorded (Figure 1), participants entered the environmental chamber to perform the first set of cognitive tests. Once completed, participants performed a 5 min warm up on the treadmill at a speed of 10 km.h^−1^ at 1% gradient, followed by the first half of the exercise protocol. The second cognitive test was performed immediately after the first half (Figure 1) in the environmental chamber. Participants moved from the chamber into a temperate environment for the 15 min rest period (Figure 1). The third cognitive test was performed after the 15 min rest period, immediately before the second half (Figure 1) in the environmental chamber. The final cognitive test was completed immediately after the second half (Figure 1) in the environmental chamber.

HR, *T*_re_, *T*_sk_, rating of perceived exertion (RPE) (Borg, 1970) and thermal sensation (TS) (Toner et al., 1986) were recorded at rest and every 5 min throughout the experimental protocol.

### Statistical analysis

Statistical analysis was conducted using IBM Statistical Package for Social Science (SPSS) (version 19). Statistical assumptions were checked using conventional graphic methods and were deemed plausible in all instances. Central tendency and dispersion are reported as the mean (± SD). All data from the main experimental trials were analyzed using a Two-Way repeated measures (condition × time) ANOVA. Bonferroni *post-hoc* comparisons were used to identify specific differences across time between changes. Two-tailed significance was accepted as *P* < 0.05.

## Results

### Physiological measures

#### Rectal temperature

**Figure 2:**
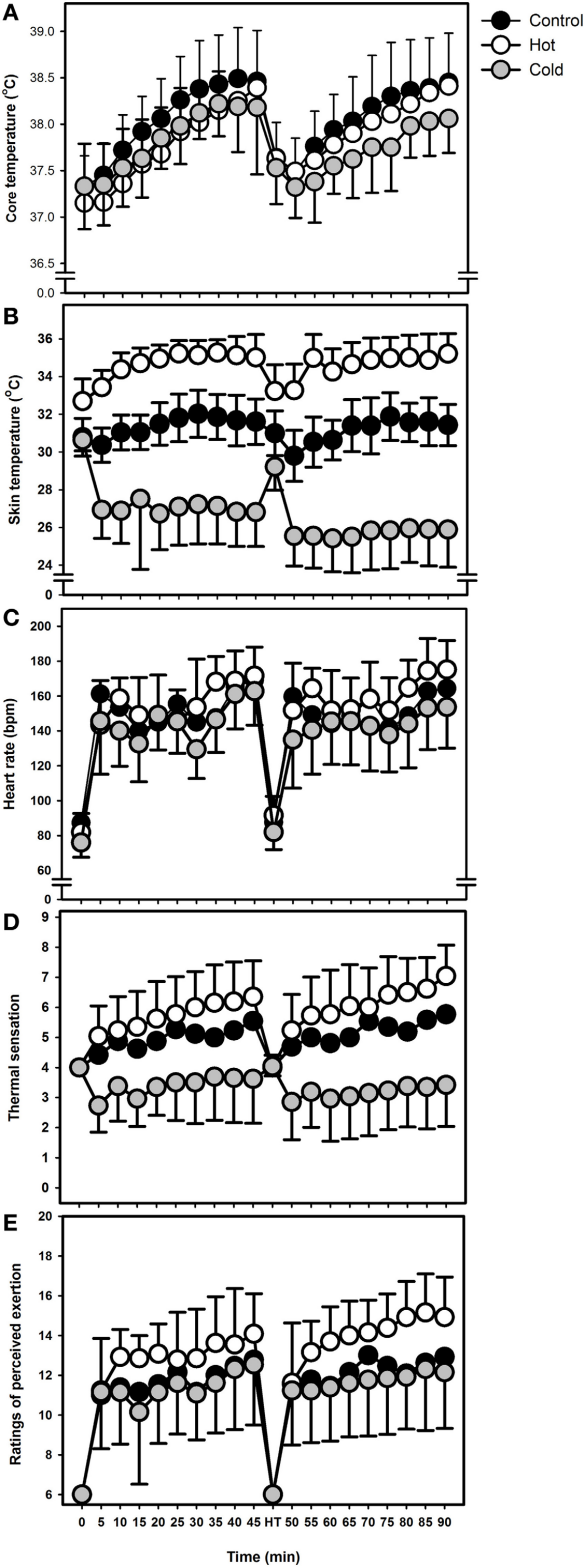
**(A)** Mean (SD) rectal temperature during the 90 min intermittent sprint exercise protocol in CONT, HOT and COLD conditions. **(B)** Mean (SD) skin temperature during the 90 min intermittent sprint exercise protocol in CONT, HOT, and COLD conditions. **(C)** Mean (SD) heart rate during the 90 min intermittent sprint exercise protocol in CONT, HOT, and COLD conditions. **(D)** Mean (SD) thermal sensation during the 90 min intermittent sprint exercise protocol in CONT, HOT, and COLD conditions. **(E)** Mean (SD) ratings of perceived exertion during the 90 min intermittent protocol in CONT, HOT, and COLD conditions.

A significant main effect for condition (*F* = 3.7, *p* = 0.039) was observed for *T*_re_ (Figure 2A), where mean *T*_re_ was 0.28°C lower in COLD than CONT (95% *CI* = 0.11 to 0.58, *p* = 0.041), however, there were no significant differences observed between CONT and HOT (mean difference = 0.20°C, 95% *CI* = −0.12 to 0.51, *p* = 0.34) or COLD and HOT (mean difference = 0.09°C, 95% *CI* = −0.21 to 0.38, *p* = 1.00). A significant interaction effect (*F* = 3.4, *p* < 0.001) showed that changes in *T*_re_ across time were not consistent across conditions, with the greatest increases in *T*_re_ being observed in HOT and the smallest increases in COLD.

#### Skin temperature

There was a significant main effect for condition (*F* = 217.5, *p* < 0.001), where mean *T*_sk_ was 3.3°C higher in HOT than in CONT (95% *CI* = 2.6 to 3.9, *p* < 0.001) and 7.8°C higher than in COLD (95% *CI* = 6.6 to 9.0, *p* < 0.001), and 4.5°C higher in CONT than in COLD (95% *CI* = 3.4 to 5.7, *p* < 0.001) (Figure 2B). A significant interaction effect also was observed (*F* = 18.6, *p* < 0.001), where *T*_sk_ in CONT remained relatively stable during the 90 min, whereas in COLD there was a 5°C reduction and in HOT there was a 3°C increase.

#### Heart rate

A significant main effect was observed for condition (*F* = 11.0, *p* < 0.001), where mean HR was 13 bpm higher in HOT than in COLD (95% *CI* = 7 to 19, *p* < 0.001), however, no significant differences were observed between HOT and CONT (mean difference = 5 bpm, 95% *CI* = −3 to 12, *p* = 0.32), or COLD and CONT (mean difference = 8 bpm, 95% *CI* = −1 to 18, *p* = 0.087) (Figure 2C). A significant interaction effect showed that the increase in HR over the 90 min was 16 bpm greater in HOT compared to COLD and CONT (*F* = 2.1, *p* < 0.001; Figure 2C).

### Subjective measures

#### Thermal sensation (TS)

A significant main effect was observed for condition (*F* = 33.9, *p* < 0.001), where mean TS was 2.4 units higher in HOT than in COLD (95% *CI* = 1.4 to 3.4, *p* < 0.001) and 1.7 units higher in CONT than in COLD (95% *CI* = 1.0 to 2.3, *p* < 0.001), but there were no significant difference between HOT and CONT (mean difference = 0.8, 95% *CI* = −0.1 to 1.6, *p* = 0.10). A significant interaction effect showed that the change in TS over the 90 min was different in the three conditions—1.8, –0.4, and 3.0 units in CONT, COLD, and HOT, respectively, (*F* = 9.1, *p* < 0.001) (Figure 2D).

#### Rating of perceived exertion

There was a significant main effect for condition (*F* = 12.0, *p* < 0.001), where mean RPE was 1.7 units higher in HOT than in COLD (95% *CI* = 0.5 to 3.0, *p* = 0.006) and 1.4 units higher than in CONT (95% *CI* = 0.2 to 2.6, *p* = 0.025), but no significant difference between CONT and COLD (mean difference = 0.4, 95% *CI* = −0.1 to 0.9, *p* = 0.20). A significant interaction effect showed that the increase in RPE across the 90 min was different across conditions, with mean increases of 6, 7, and 9 units for the COLD, CONT, and HOT conditions, respectively (*F* = 3.2, *p* < 0.001) (Figure 2E).

### Cognitive performance

#### Dual task

**Figure 3:**
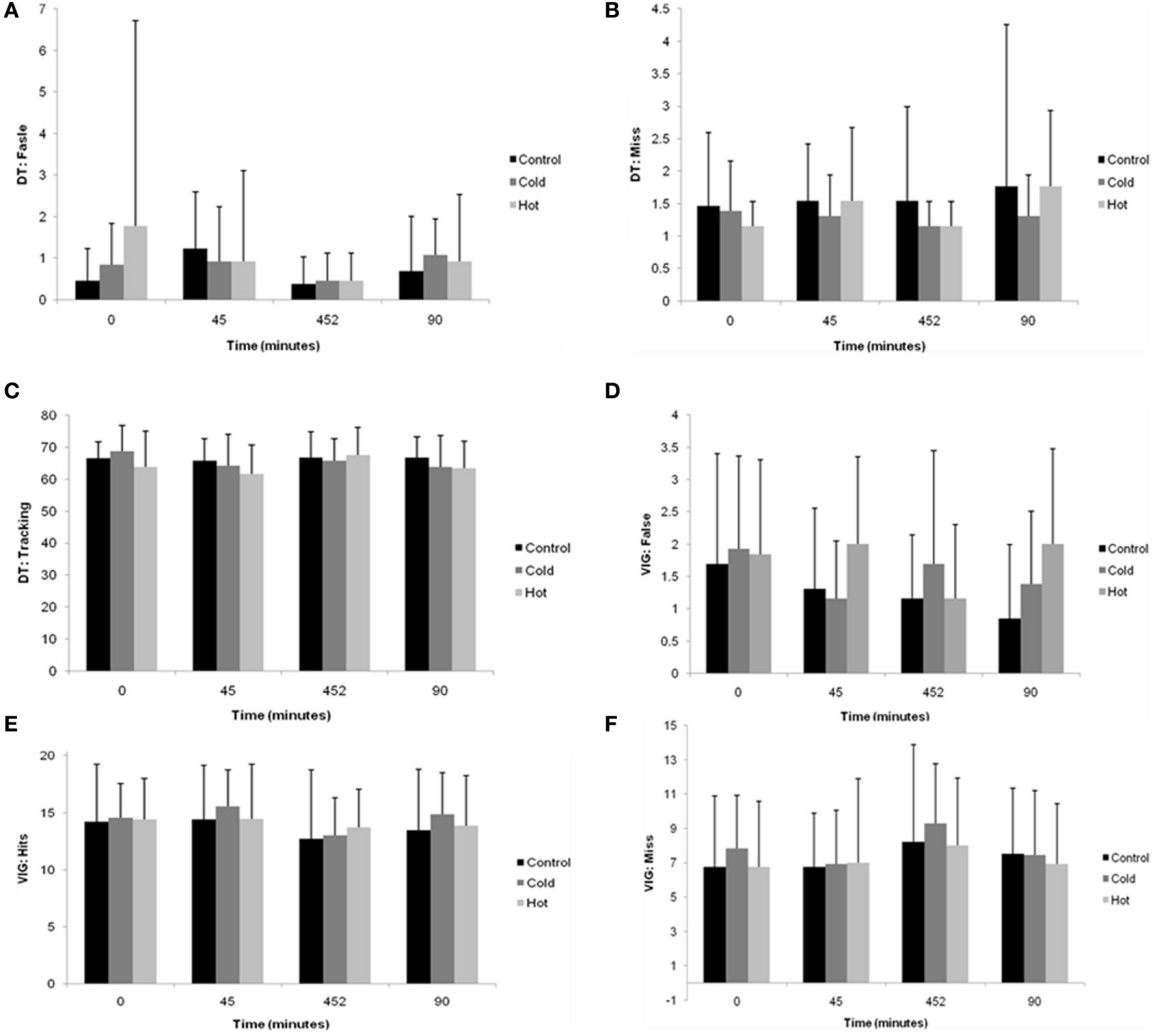
**Mean (SD) dual task scores during the 90 min intermittent sprint exercise protocol in CONT, HOT, and COLD conditions (A) FALSE, (B) MISS, and (C) TRACKING.** Mean (SD) vigilance scores during the 90 min intermittent sprint exercise protocol in CONT, HOT, and COLD conditions **(D)** FALSE, **(E)** HITS, and **(F)** MISS.

There were no significant main effect for condition (*F* = 0.2, *p* = 0.79), time (*F* = 1.9, *p* = 0.15), or an interaction effect between condition and time (*F* = 1.0, *p* = 0.46) observed in FALSE scores (Figure 3A).

There was no statistically significant main effects of condition (*F* = 0.2, *p* = 0.81) and time (*F* = 0.8, *p* = 0.52), or condition × time interaction (*F* = 0.6, *p* = 0.72) observed for MISSED scores (Figure 3B).

For TRACKING scores, there were no statistically significant main effects of condition (*F* = 1.9, *p* = 0.18) and time (*F* = 0.8, *p* = 0.49), or condition × time interaction (*F* = 1.4, *p* = 0.22) observed (Figure 3C).

#### Vigilance

There were no statistically significant main effects of condition (*F* = 1.5, *p* = 0.24) and time (*F* = 2.1, *p* = 0.11), or condition × time interaction (*F* = 1.2, *p* = 0.31) observed for FALSE scores (Figure 3D).

There was no statistically significant main effects of condition (*F* = 0.6, *p* = 0.54) and time (*F* = 0.3, *p* = 0.84), or condition × time interaction (*F* = 1.6, *p* = 0.15) observed for HITS scores (Figure 3E).

No statistically significant main effects for MISSED scores were observed in condition (*F* = 0.5, *p* = 0.63) and time (*F* = 0.2, *p* = 0.92), or condition × time interaction (*F* = 1.6, *p* = 0.17; Figure 3F).

Since the cognitive performance effects were small and not statistically significant, mean differences and associated confidence intervals for pairwise comparisons have been omitted; however, an appreciation of pairwise mean differences can be obtained from Figure 3.

## Discussion

The aim of this investigation was to examine whether environmental conditions typical of the UEFA region and season, could have an effect on decision making and physiological responses within participants during a soccer specific intermittent sprint protocol (Drust et al., 2000). The main finding was that HOT caused no detriment to decision making performance, thus rejecting the hypothesis that heat stress would cause decrement to referee cognitive performance. Similar results were observed for COLD as hypothesized.

The novel finding from the present study is that thermal challenges (HOT and COLD) do not negatively affect referees decision making ability—negative cognitive responses were not increased during exposure to hot conditions. Moreover, positive cognitive responses suffered no decrement during exercise in the heat. Similarly, no difference in core temperature was observed between conditions [CONT and HOT conditions (*p* = 0.33); COLD and HOT conditions (*p* = 1.00)]. A lack of difference in *T*_re_ between conditions may be attributable to a number of variables; it may be that a lower resting *T*_re_ prior to exercise in HOT may have masked any difference in *T*_re_ increase rate across conditions. Moreover, it may be that the exercise protocol was not strenuous enough to induce sufficient thermoregulatory strain in the hot condition. Finally, it may be HOT was only warm conditions, again providing insufficient thermoregulatory load. However, *T*_sk_ was significantly higher in HOT than CONT (*p* < 0.001) and COLD (*p* < 0.001).

The absence of significant differences in cognitive performance between conditions within the present study (see Figure 3) are likely attributable to the lack of significant differences in *T*_re_ observed [CONT (38.05 ± 0.08) vs. HOT (37.85 ± 0.06); *p* = 0.33; COLD (37.7 ± 0.09) vs. HOT (*p* = 1.00) (see Figure 2A)]. Exercise-heat stress induced increases in core temperature results in a reduction in cerebral blood flow (Fujii et al., 2008; Brothers et al., 2009; Hayashi et al., 2011), suppressed arousal (Nielsen et al., 2001) and reductions in cognitive capacity (Bandelow et al., 2010). Moreover, reductions in cerebral blood flow have been shown to correlate with exercise fatigue (Nybo and Nielsen, 2001; Thomas and Stephane, 2008). This interplay between core body temperature and cognitive processes (Nielsen et al., 2001; Nybo and Nielsen, 2001; Bandelow et al., 2010), which are not significantly disturbed within the utilized experimental design (likely due to the exercise protocol used (Drust et al., 2000), as discussed later in text), may provide explanation for the lack of variance in cognitive performance across and between conditions in the present study.

The utilized “Drust Protocol” (Drust et al., 2000) provides an instrument which is designed to mimic the movement patterns (various speeds and durations of movements and associated rest periods) of out-field soccer players. As soccer players and referees display similar types of movement (walk, jog, run, cruise and sprint) and cover similar distances [referees 9–13 km (Reilly and Gregson, 2006); players ~ 9–12 km (Di Salvo et al., 2013)] over the duration of a 90 min match, the Drust Protocol (Drust et al., 2000) was selected for the present study. This protocol is conducted on a motorized treadmill, using fixed speeds which are not individualized to each participant's physiological capacity, e.g., peak sprint/treadmill speed. Lack of individualization in physiological load, likely produced differential internal and external loads within subjects. The mean average HR across conditions in the present study (COLD: 138 ± 5; CONT: 146 ± 6; HOT: 151 ± 5), compared to 165 bpm from soccer match play (Catterall et al., 1993) and 167 bpm from an appropriately valid and reliable treadmill based soccer simulation (Aldous et al., 2013) respectively, suggests that physiological load in the present study was not indicative, thus not valid, in light of the values typically seen in soccer match play (Catterall et al., 1993; Castagna et al., 2007; Krustrup et al., 2009; Bradley and Noakes, 2013). Further concern is raised by the highest mean RPE recorded throughout HOT (30°C) in the present study being 15 (HARD), whereas repeated sprint protocols typically elicit RPE >18 (Drust et al., 2005).

Additionally, match analysis data (Reilly and Gregson, 2006) provides commentary on the distances covered by soccer match officials, the types of movement, and the difference in performance between the first and second half. It was reported that soccer referees from different national leagues (English Premier League & Danish SuperLiga) (Catterall et al., 1993; Krustrup and Bangsbo, 2001) as well as elites soccer players (Mohr et al., 2003; Bradley and Noakes, 2013), all covered less distance running at high intensities and less overall distance, during the second half of matches. As the Drust protocol utilizes fixed speeds on a motorized treadmill there is no allowance for this bi-phasic performance between halves, and thus not reflective of actual match play performance in soccer referees (Catterall et al., 1993; Krustrup and Bangsbo, 2001) or players (Mohr et al., 2003; Bradley and Noakes, 2013). The HOT condition final *T*_re_ (90 min) (38.4°C ± 0.4) was lower than that reported in soccer match play data for both professional soccer players (38.5°C) and recreational soccer players (39.0°C) (90 min); even though these results were collected in temperate conditions (16°C) (Edwards and Clark, 2006). This lack of appropriate specific physiological load (i.e., reflective of a referee/player during a game), could be pivotal in the lack of cognitive impairment seen in the present study, for example the core temperature cognition nexus previously described, which was hypothesized to occur within HOT. As the movement patterns of soccer referees and players are similar (Weston et al., 2011) such comparisons are possible, and demonstrate that the physiological load experienced during the Drust Protocol was not sufficient, nor valid when compared to match play data (Mohr et al., 2003; Barros et al., 2007; Bradley and Noakes, 2013).

A further point of interest that may explain the disparity in continuity between the present study and that of Drust et al. (2000) may be the difference in fitness between the two studies. The study by Drust et al. (2000) used university standard football players where as the present study used both semi-professional footballer players and referees. If those participants recruited for the present were fitter than those from Drust et al. (2000) due to their superior level of soccer participation it may be that the Drust protocol is valid for use on amateur soccer players but not semi-professional/professional. In the present study there were no significant differences in decision making ability during and between all conditions, likely due to the validity issues outlined above regarding the employed protocol (Drust et al., 2000).

A recently published protocol utilizing a non-motorized treadmill based intermittent soccer-specific performance test (iSPT) (Aldous et al., 2013) provides the individualized variance (e.g., reduction in distance covered between 1st and 2nd halves) observed in real-life match play in soccer players (Mohr et al., 2003; Barros et al., 2007; Bradley and Noakes, 2013) and referees (Catterall et al., 1993; Krustrup and Bangsbo, 2001), which was absent in the utilized protocol (Drust et al., 2000). Data from the iSPT displays reductions in total distance covered and distance covered at high intensities during the second 45 min of a 90 min protocol (Aldous et al., 2013), similar to the field data observed in elite referees (Krustrup and Bangsbo, 2001; Reilly and Gregson, 2006) and players (Mohr et al., 2003; Barros et al., 2007; Bradley and Noakes, 2013). All variables associated with internal and external load in response to iSPT (Aldous et al., 2013), were shown to be reliable, and valid in comparison to match play data. Therefore, it is recommended that the present experimental design be repeated with the protocol employed (Drust et al., 2000) replaced with iSPT (Aldous et al., 2013), or a similar appropriately constructed protocol (Williams et al., 2010).

The interaction between skin blood flow (*T*_sk_) and *T*_re_ must not be overlooked. Alterations in skin blood flow may explain why *T*_re_ did not display noticeable change between conditions. It is plausible that increases in skin blood flow and thus increases in skin temperature provided sufficient thermoregulatory homeostasis via heat evaporation and radiation that *T*_re_ did not increase in the hot condition—particularly if either the hot condition was not hot enough, the exercise protocol was not strenuous enough or a combination of both. This provides explanation for the non-significant difference in *T*_re_ between conditions as well as the statistically significant difference in *T*_sk_ between conditions.

As hypothesized, there was no effect on cognition in the COLD condition. Whilst performing the exercise protocol, metabolic heat would be produced by the working muscles (Brotherhood, 2008) and this may serve to maintain deep body temperature (*T*_re_). Whilst maintaining *T*_re_, there will be a decline in temperature in the peripheral tissue (Tipton, 2006), which was observed in the present investigation, as *T*_sk_ was significantly lower in the COLD condition compared to both CONT and HOT. Though the experimental hypothesis was proven correct with regard to COLD, the formulation of the employed protocol (Drust et al., 2000), as discussed previously, may confound the validity of the observed data and its interpretation. Unpublished data from our laboratories suggest that the effects of extreme environmental conditions on decision making of non-exercising participants (e.g., goal line officials employed within the UEFA region) causes a reduction in cognitive performance in a similar COLD condition, with said performance unaffected in a warmer conditions, as observed in other previous research (Mäkinen et al., 2006). Evidently, the interaction of exercise, cognition and environmental stress is multifaceted, and requires further elucidation, specifically regarding environments of greater stress than those used within the present study.

Aside from the environment mediated challenge to cognition processes, it is possible that cognitive function may be impaired during by exercise *per se* (McMorris and Graydon, 1997). Previous research has found that neural activation associated with a task, whilst disturbed during task (e.g., exercise), rapidly returns to baseline levels after exercise (Magnié et al., 2000). Such in-task disturbance is likely due to a large part of the brain being associated with basic sensory processes and motor outputs, and thus, must come at the expense of other neural tasks (Dietrich and Sparling, 2004). Therefore, future work, utilizing an appropriate protocol as suggested (Williams et al., 2010; Aldous et al., 2013), should seek to assess decision making ability during the exercise task itself. To enhance ecological validity further the use of a soccer specific decision making tool should be considered for future research. The tool used in the present study (PSCHE Software) was developed for use in a clinical setting. And although it provides useable, quantitative data it has not been employed extensively in a sporting context. It may be that a soccer specific tool more sensitive than the PSYCHE software would highlight differences in decision making that were not observed in the present study.

In conclusion, intermittent sprint exercise in any condition did not have a significant effect on decision making ability of the participants. The exercise protocol utilized (Drust et al., 2000), as described, likely did not provide a truly indicative simulation of the internal and external load of soccer match play, and thus referee movement patterns. This lack of reflective internal and external loads resulted in the tripartite relationship between environmental UEFA region and season specific environmental stress, cognition processes and exercise not being securely and fully elucidated within the utilized experimental design. Future work should look to assess cognition during exercise/game play in conjunction with the use of an appropriately constructed protocol (e.g., Aldous et al., 2013) in a variety of environmental conditions to enhance ecological/external validity to not only the UEFA region, but that of Federation Internationale de Football Association (FIFA).

## Funding

This was a Union of European Football Associations (UEFA) funded project.

### Conflict of interest statement

This original research was funded by the Union of European Football Associations (UEFA). The authors declare that the research was conducted in the absence of any commercial or financial relationships that could be construed as a potential conflict of interest.
